# Exosome-mediated delivery of miR-30a sensitize cisplatin-resistant variant of oral squamous carcinoma cells via modulating Beclin1 and Bcl2

**DOI:** 10.18632/oncotarget.27557

**Published:** 2020-05-19

**Authors:** Bhagyashri Kulkarni, Piyush Gondaliya, Prathibha Kirave, Rakesh Rawal, Alok Jain, Rachana Garg, Kiran Kalia

**Affiliations:** ^1^Department of Biotechnology, National Institute of Pharmaceutical Education and Research, Ahmedabad, Gujarat, India; ^2^Department of Life Science, Gujarat University, Ahmedabad, Gujarat, India; ^*^These authors contributed equally to this work

**Keywords:** chemoresistance, miRNA30a-5p, exosomes, oral cancer, autophagy

## Abstract

Exosomes facilitate cross-talk amongst tumor cells, and thus also possess the potential to influence tumor-microenvironment and chemo-resistance. miRNAs, the important constituent of exosomes, are often dysregulated in cancer. They have been shown to play an essential role in tumor progression, metastasis, invasion, and resistance developed against different therapies. Acquisition of cisplatin-chemoresistance remains a major hurdle in the effective treatment of oral squamous cell carcinoma (OSCC). In this study, we demonstrate the importance of exosome-mediated miR-30a transfer in conferring cisplatin sensitivity in the otherwise resistant OSCC cells. Notably, miR-30a was found to be significantly reduced in exosomes isolated from the serum of OSCC patients, especially those having disease-recurrence, post cisplatin treatment. In conjunction with the findings in clinical samples, decreased miR-30a expression was observed *in vitro* in the cisplatin-resistant cultured OSCC cells compared to the cisplatin-sensitive cells. Besides, we identified *Beclin1*, an autophagy-related marker, as a target of miR-30a and found it to be overexpressed in cisplatin-resistant OSCC cells, thus indicating at its possible negative-regulation by miR30a. Exosomes from the cisplatin-resistant cells that have been transfected with miR-30a mimics, when delivered to the naïve cisplatin-resistant cells, caused not only the significant enhancements in miR-30a expression but also a concomitant decrease in Beclin1 and Bcl2 expression (autophagic and anti-apoptotic marker). More importantly, this together resulted in the sensitization of cisplatin-resistant cells. Thus, our study highlighted the role of exosomal-mediated miR-30a transfer in regaining sensitivity of the cisplatin-resistant OSCC cells via Beclin1 and Bcl2 regulation and hence suggests at its potential therapeutic role.

## INTRODUCTION

Oral squamous cell carcinoma (OSCC) is the sixth most frequent cancer that affects the human population worldwide [1]. Despite significant developments in the current treatment modalities for OSCC, the disease-free survival rate has remained poor for the last several decades. Cisplatin, though is a first-line chemotherapeutic drug used for the treatment of OSCC patients [2, 3], acquisition of cisplatin-resistance to date remains a major impediment in its efficacious clinical application. There is, therefore, an urgent need to better understand the underlying mechanisms of cisplatin resistance as well as identifying reliable targets/mediators which can facilitate the restoration of sensitivity towards cisplatin.

Exosomes (the membrane secretory vesicle) acts as cargo for various molecular content (miRNA, LncRNA, msRNA, DNA, protein, enzyme) that depend on their origin and status; exosomes thus mediate cell-cell communication and convey biological signals to regulate cellular processes [4]. Emerging studies have shown that cancer cells release exosomes at a higher rate compared to the normal cells, and contribute significantly in instituting a milieu that supports neoplastic proliferation, angiogenesis, invasion, and metastasis [5]. In the past decade, the role of tumor cell derived-exosomes in inhibiting immune-surveillance and hence bestowing oncogenic transformation has gathered increasing attention [6]. They have also been implicated in the development of drug-resistance [7], and therefore now being considered as promising candidates both for cancer diagnostics and therapeutics [8].

An increasing amount of evidence has indicated that exosomes-derived miRNA plays an essential role in mediating cellular crosstalk within tumor-microenvironment [4], therefore are capable of moderating tumor progression and chemoresistance in many cancer types [7, 9]. Pioneering studies have shown the role of pancreatic cancer-derived exosomes in transferring miRNAs (miR212-3p, miR 203, mir 21) to dendritic cells leading to enhanced invasiveness and drug resistance [10–13]. Exosome-mediated miR155 delivery was found to cause EMT and associated chemoresistance in breast cancer [14]. On the other hand, exosome-mediated delivery of miR-151a and miR-128-3p has been shown to sensitize glioblastoma and colorectal cancer cells to temozolomide and oxaliplatin respectively [15–18].

miRNAs have also been shown to moderate chemoresistance via regulating autophagy [19], an approach adopted by tumor cells to survive the metabolic stress imposed by tumor-microenvironment [20, 21]. miR-30a has been shown to play a role in mediating cisplatin-chemoresistance in melanoma and pancreatic adenocarcinoma *via* IGFR1 and SNAI1 [17, 18]. However, in some other studies, the reverse role of miR30a on drug-resistance has been documented. Zou et al. showed a significant reduction in miR-30a expression in HeLa, MCF7 and HepG2 cancer cells following cisplatin treatment, whilst the forced expression of miR-30a sensitizes them to cisplatin *via* blocking beclin 1-mediated autophagy [22]. Similarly, miR30a induction in cisplatin-resistant lung cancer cells led to inhibition of Beclin 1-mediated autophagy and a concomitant increase in apoptosis [23]. The author thus suggested that enhancing miR-30a expression in breast, liver, and lung tumor cells offers a promising approach to combat chemoresistance. Besides, miR-30a has proven its function in regulating epithelial-mesenchymal transition, impeding proliferation and metastasis in multiple cancers and autophagy in CML [24]. Notably, reduced expression of miR-30a has recently been reported in OSCC, and it has also been associated with decreased cell proliferation, migration, and invasion [25, 26]. Cisplatin chemoresistance is also a big hurdle in OSCC treatment. Hence, it is worth determining if exogenously increasing miR-30a in OSCC has any role in combating cisplatin chemoresistance, regulating autophagy, a process known to influence chemoresistance and if exosomal-mediated miR30a delivery can be exploited as an approach to enhance the therapeutic efficacy.

In the present study, we show significantly decreased expression of miR-30a in oral cancer patients with disease recurrence post cisplatin treatment and OSCC cultured cells having cisplatin resistance. Herein, we present the first evidence that exosomal-mediated miR-30a delivery in the cisplatin-resistant OSCC cells led to decreased autophagic response *via* Beclin1 while it augments apoptosis by inhibiting Bcl2, hence mediating reversal of cisplatin sensitivity. Our results thus establish the significance of exosome-mediated miR-30a delivery in combating chemoresistance in oral cancer cells and open up new avenues for designing of exosomes-based clinical management of oral cancer.

## RESULTS

### Beclin1 acts as a target for miR-30a in OSCC

**Figure 1:**
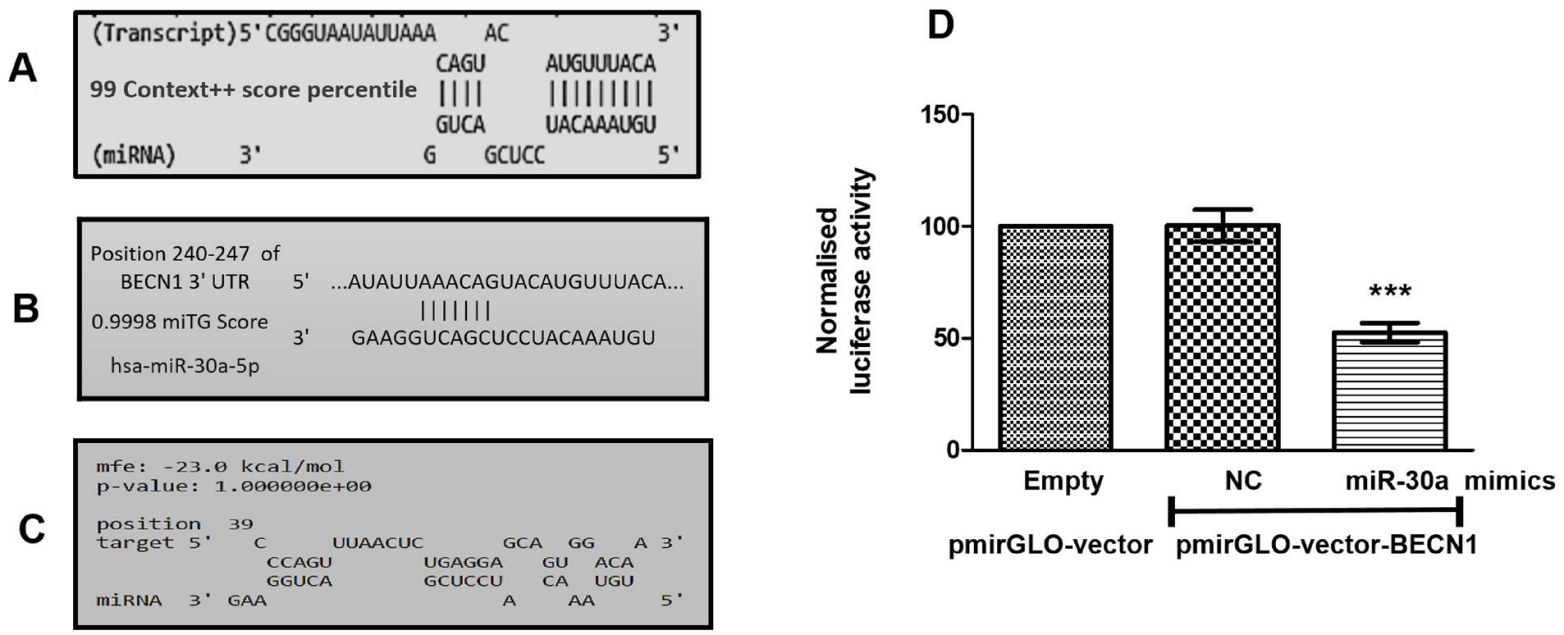
(**A**) Binding position prediction of miR-30a with Beclin1 using TargetScan web-based tool. (**B**) Binding position prediction of miR-30a with Beclin1 using the DIANA microT-CDS tool. (**C**) Binding energy prediction of miR-30a with Beclin1 by RNAhybrid. (**D**) BECN1 luciferase activity in cis^Res^ cells co-transfected with either empty vector or pmirGLO-Becn1 vector having miR-30a target sequence and either NTC or miR30a mimics. Data are expressed as the mean +/– SD. ^***^ *P* < 0.001, significant difference vs. control group (*n* = 3). Two independent experiments gave similar results.

Using three web-based tools, we predicted the gene targets of miR-30a in OSCC. TargetScan and DIANA-micro-T-CDS predicted 431 and 1782 gene-targets respectively (data not shown). Out of these, the top 100 gene-targets selected from targetscan (based on total context ++ score percentile) and DIANA-micro-T-CDS (based on miTG score) were considered for further screening. Next, following their known implication in cancer chemoresistance, 21 gene-targets were further shortlisted (Supplementary Table 1). Notably, *Beclin1*, an autophagy marker, showed the highest score with both TargetScan and DIANA-micro-T-CDS predictions (Supplementary Table 1 and Figure 1A and 1B). We further validated the selected gene-target using RNAhybrid tool wherein minimum free energy of binding of *Beclin1* with miR-30a was found to be –23 kcal/mol (Figure 1C). Finally, based on the consensus obtained from all the three web-based tools, *Beclin1* was selected as a potential target for miR-30a in cisplatin-resistant OSCC.

We next validated *in-silico* predictions in cultured cisplatin-resistant OSCC cells using a luciferase reporter assay. Of note, mimics-mediated forced-expression of miR-30a in cis^Res^ cells significantly decreased the *miR-30a* -3′UTR-*Beclin1* luciferase activity compared to the cis^Res^ cells transfected with *NTC* or empty vector control, thus demonstrating miR-30a-mediated regulatory control of *Beclin1* (Figure 1D).

### Exosomal miR-30a is downregulated, while Beclin1 is up-regulated in cis^Res^ OSCC cells and patients

**Figure 2:**
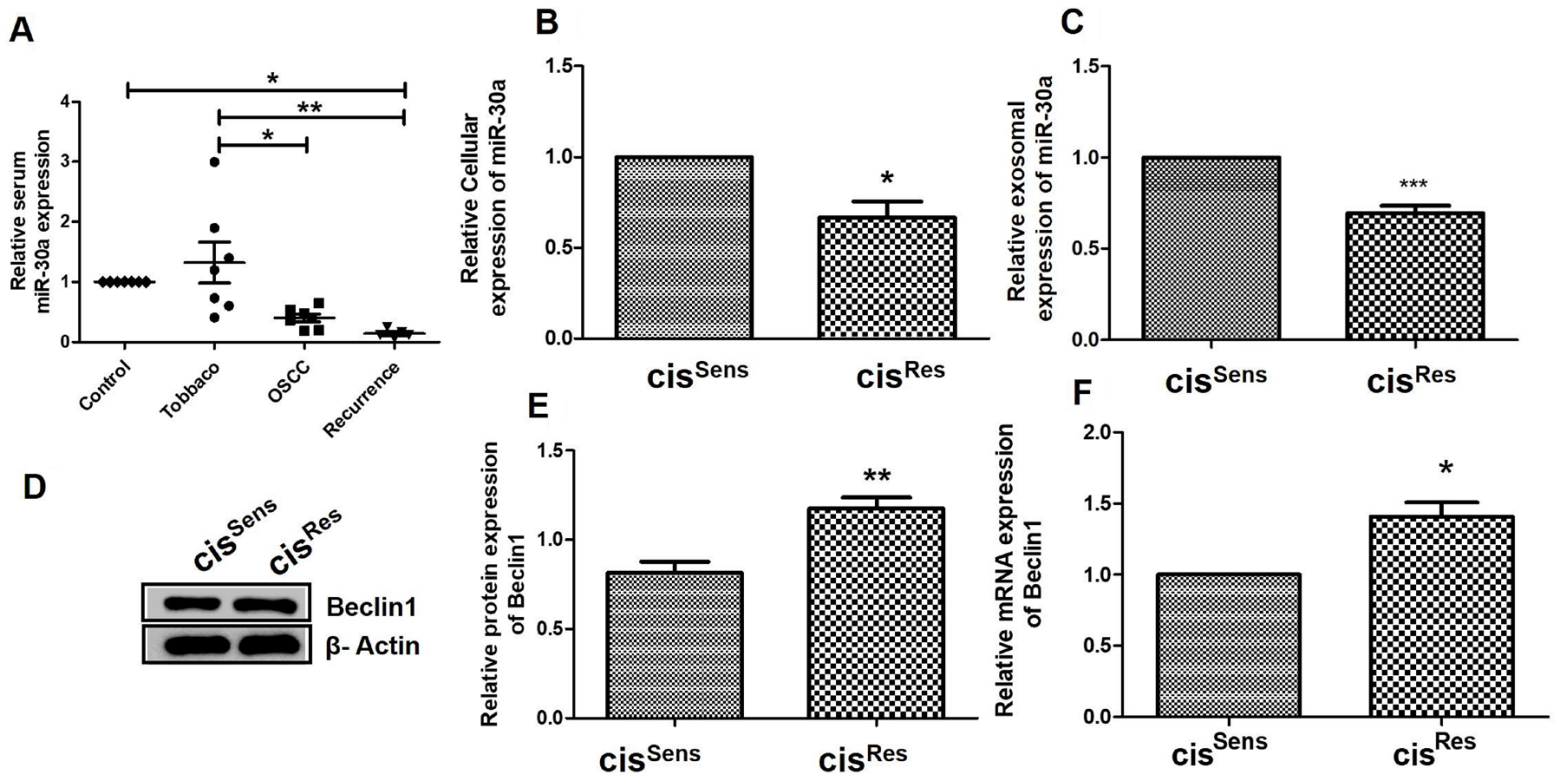
(**A**) miR-30a expression in clinical samples was quantified by qRT-PCR and normalized to U6 as a housekeeping gene. miR-30a expression profiling in cis^Res^ and cis^Sens^ oral cancer cells at the (**B**) total cellular and (**C**) exosomal level. (**D**) Western blot analysis for Beclin1 expression in cis^Res^ and cis^Sens^ oral cancer cells. (**E**) Densitometry of Beclin 1 Western blot normalized to actin as the loading control. (**F**) Beclin 1 mRNA expression as analyzed by qRT-PCR. 18S was used as a housekeeping gene.

We next determined if miR-30a has any role in acquired cisplatin-resistance in OSCC. Interestingly, miR-30a expression was found to be significantly reduced in exosomes isolated from the serum of OSCC patients *vs* healthy volunteers (Figure 2A). Exosomes from tobacco smokers showed aberrant miR-30a expression compared to healthy volunteers. Of special relevance, is the highest and significant reduction in miR-30a expression observed in the exosomes from OSCC patients with tumor recurrence post cisplatin treatment *vs* healthy controls. We next corroborated our finding in clinical samples depicting an association of reduced miR-30a levels with disease recurrence, *in vitro* using cis^Res^ OSCC cells. Notably, as observed in OSCC patients, both cellular and exosomal miR-30a expression was significantly lower in cis^Res^ OSCC cells compared to cis^sens^ cells (Figure 2B and 2C).

We further evaluated the importance of miR-30a in cis^Res^ OSCC by measuring the expression of its target gene, *beclin1* (Figure 1). In concordance with the reduced miR-30a expression, cis^Res^ cells showed enhancements in Beclin1 (an autophagy marker) both at the protein (Figure 2D, 2E) and mRNA levels (Figure 2F), when compared to the cis^Sens^ cells.

### miR-30a restoration inhibited Beclin1 expression and conferred cisplatin sensitivity in cis^Res^ OSCC cells

**Figure 3:**
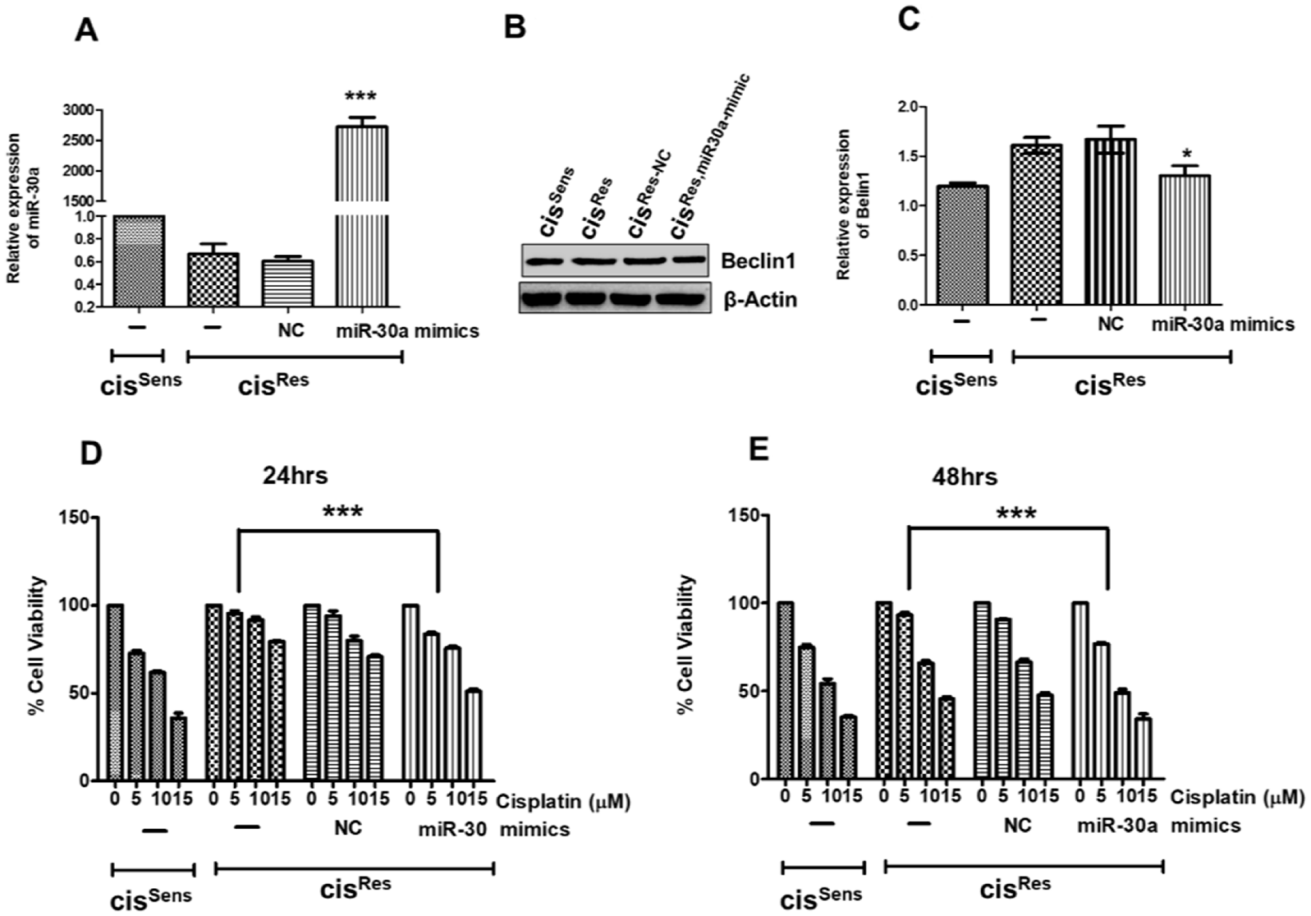
cis^Res^ oral cancer cells were transfected with either miR-30a mimics or NC (negative control) and after 24 h, the following effects were analyzed. cis^Sens^ cells were employed for the comparison. (**A**) miR-30a expression as measured by qRT-PCR. (**B**) Beclin1 protein expression as determined by the Western blot analysis. (**C**) Densitometry analysis of Beclin1 Western blot was carried out by normalization to β-actin employed as the loading control. Following miR30a restoration, cells were treated with different concentrations of cisplatin. Cell viability was measured by MTT assay after **(D**) 24 h and (**E**) 48 h. Error bars represent the mean ± SD of three independent experiments. ^*^ *p* < 0.05, ^***^ *p* < 0.001.

We identified *Beclin1* as a miR-30a target in OSCC (Figure 1). However, its association with cisplatin-resistance in OSCC thus far remains undetermined. Hence, we next investigated if the decreased expression of miR-30a also modulates Beclin1 in cis^Res^ OSCC cells. For this, using a mimics-transfection approach, we first restored the miR-30a expression in cis^Res^ OSCC cells (Figure 3A). Notably, miR-30a restoration in cis^Res^ cells (referred hereafter as Cis^Res, miR-30a-mimic^) significantly decreased the Beclin1 expression (Figure 3B, 3C). cis^Sens^ cells were employed in the study as a control.

We next evaluated if the observed miR-30a effect on autophagy marker influences OSCC cell chemosensitivity. Remarkably, mimics-mediated miR-30a restoration in cis^Res^ OSCC cells resulted in regaining of their cisplatin sensitivity when compared to the cells transfected with *NC* (Figure 3D, 3E). Notably, cisplatin resistance was significantly reduced at all tested doses (0–15 μM) and time-points (24–48 h). The IC_50_ value of cis^Res-30a-mimics^ cells was found to decrease by two-fold compared to cis^Res^ cells. This regains in cisplatin-sensitivity is comparable with the parental cis^Sens^ OSCC cells, especially at 48 h time-point. Our results thus argue for miR-30a implication in conferring a cisplatin-sensitive phenotype to the chemo-resistant OSCC cells.

### miR-30a overexpression induces apoptosis in cis^Res^ OSCC cells

**Figure 4:**
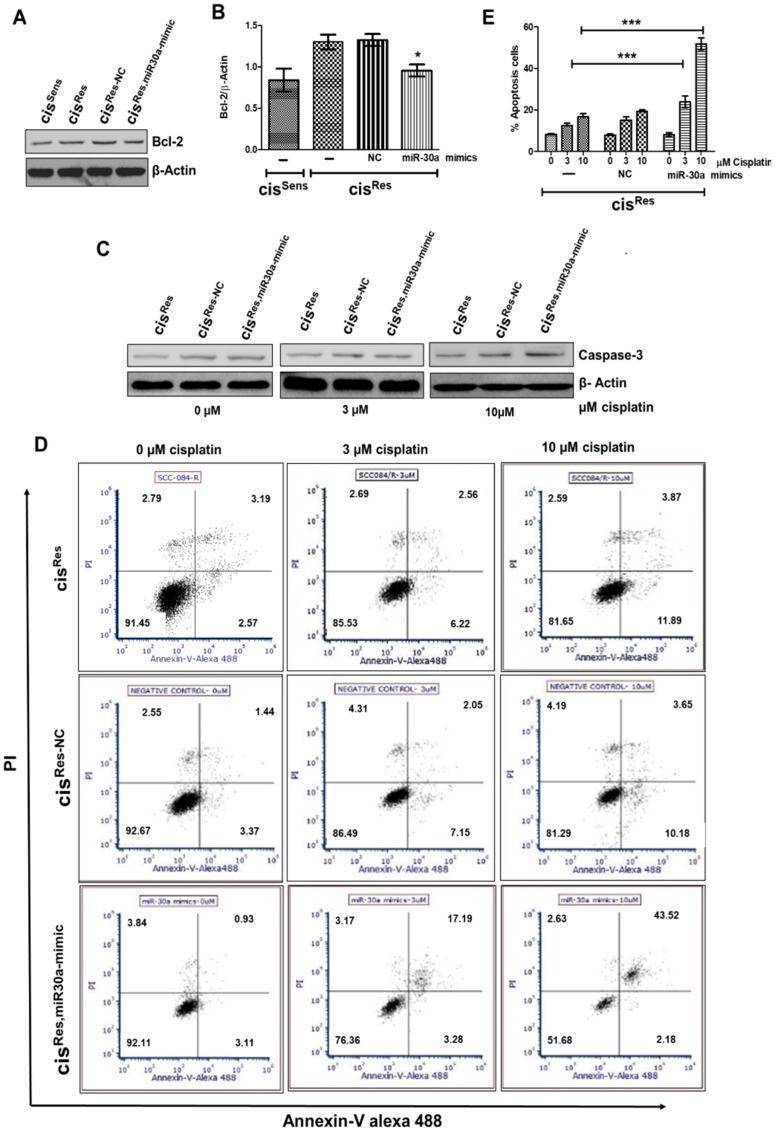
cis^Res^ oral cancer cells were transfected with either miR-30a mimics or NC (negative control) and after 24 h, the following effects were analyzed. cis^Sens^ cells were employed for the comparison. (**A**) Bcl2 protein expression by Western blot. (**B**) Densitometry analysis of Bcl2 Western blot normalized to β-actin as the loading control. (**C**) Mimics or NC transfected cells were treated with or without cisplatin and caspase-3 expression was analyzed by Western blot analysis. (**D**) Cells were treated as described in (C) and after 72 h of cisplatin treatment, cells were collected and stained with FITC-conjugated annexin-V and PI and analyzed by flow cytometry. (**E**) Graphical representation of the percentage of apoptotic cells as analyzed by FACS analysis. Error bars represent the mean ± SD of three independent experiments. ^*^ *p* < 0.05, ^***^ *p* < 0.001.

*BCl2* has been documented as a miR-30a target in various cancers, including non-small cell lung carcinoma, renal carcinoma, cutaneous squamous cell carcinoma [27, 28]. However, their association in OSCC remains elusive. Herein, we show that mimics-mediated miR-30a restoration in cis^Res^ OSCC cells led to downregulation of anti-apoptotic protein Bcl2, while it enhanced the caspase3 expression compared to *NC* transfected cells (Figure 4A–4C); increment in caspase3 expression followed cisplatin dosage. miR-30a role in conferring an apoptotic phenotype was further confirmed by Annexin-V-PI staining. As expected, majority of the cis^Res^ OSCC cells were viable (81–90%) even after treatment with two increasing concentrations of cisplatin (3 and 10 μM) (Figure 4D, 4E). Comparable results were obtained in *NC*-transfected cis^Res^ cells (cis^Res-NC^). Of note, when cis^Res-30a-mim^ OSCC cells were treated with increasing concentrations of cisplatin, 76–51% of Annexin V^–^PI^–^ cells were observed. An increase in Annexin V^+^PI^+^ population was observed in cells receiving cisplatin treatment (3 or 10 μM, 17.19 and 43.52% respectively), indicative of late apoptotic or dead cells. Our results thus indicate that miR-30a restoration activates apoptosis in cis^Res^ OSCC cells.

### miR-30a restoration impedes migration of cis^Res^ OSCC cells

**Figure 5:**
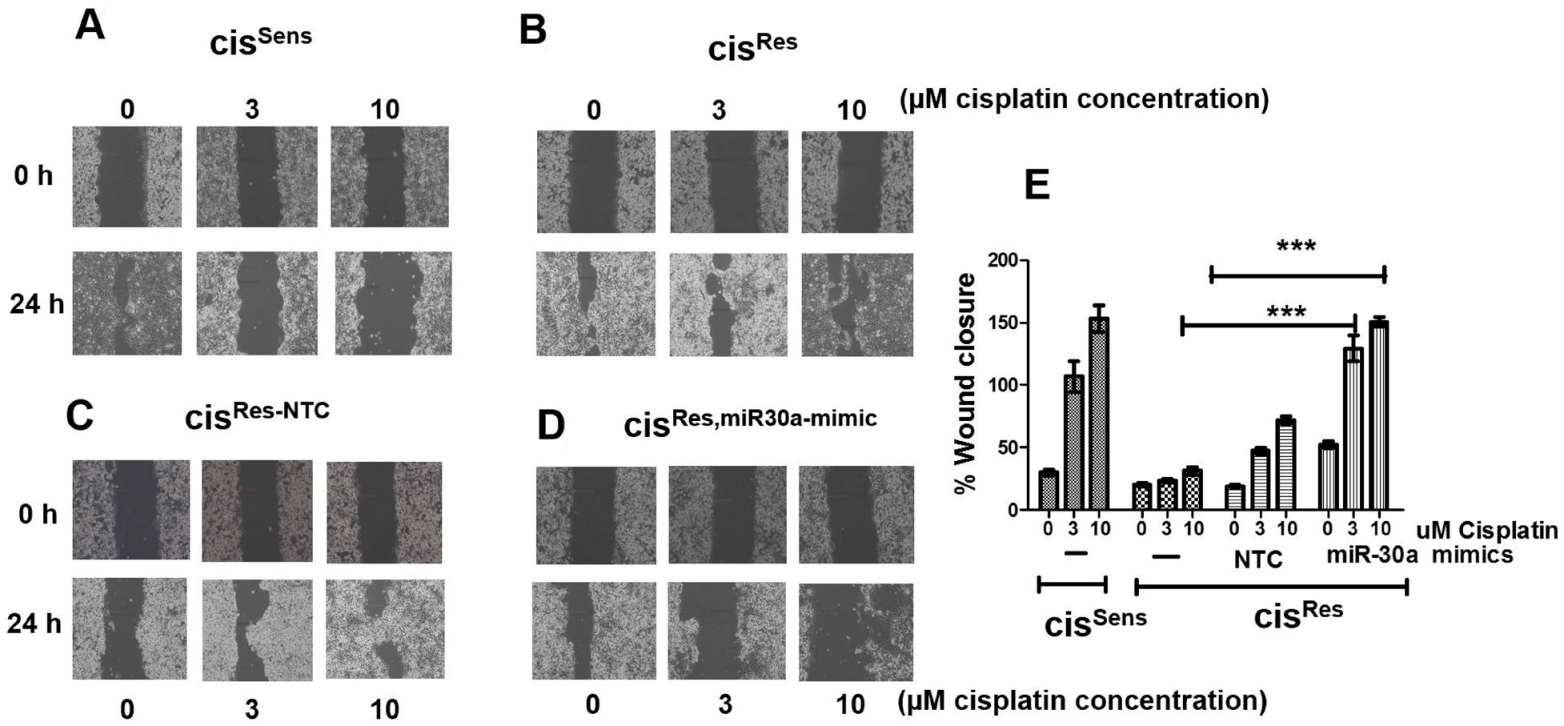
The effects of cisplatin treatment were analyzed on the migration of cis^Res^ oral cancer cells transfected with either miR-30a mimics or NC (non-target control) by wound assay. cis^Sens^ cells were employed for the comparison. (**A**–**D**) Representative images of wound closure taken at 0 and 24 h after the scratch was made and cisplatin treatment was given. (**E**) quantification of wound closure as analyzed using Image J. Data are expressed as mean ± SD. ^***^ *p* < 0.001.

Numerous studies have indicated that the development of chemoresistance influences cancer metastasis [29]. Having observed the effect of miR-30a on cisplatin-sensitization, we next intended to determine if miR-30a restoration modulates the migratory potential of cis^Res^ OSCC cells. As analyzed by wound assay, cisplatin treatment in a dose-dependent manner enhanced the migration of cis^Res^ OSCC cells, as evident from the faster wound-closure, when compared to cis^Sens^ cells (Figure 5). The wound closure percentage is comparable in cis^Res-NC^ and cis^Res^ OSCC cells. Notably, little or no closure was observed in the cis^Res-30a-mim^ OSCC cells at all the cisplatin concentration tested, thereby suggesting that forced expression of miR-30a impaired the motile characteristic of cis^Res^ cells.

### Exosome-mediated miR-30a delivery in cis^Res^ OSCC cells modulated their autophagic, apoptotic and chemo-sensitive response

**Figure 6:**
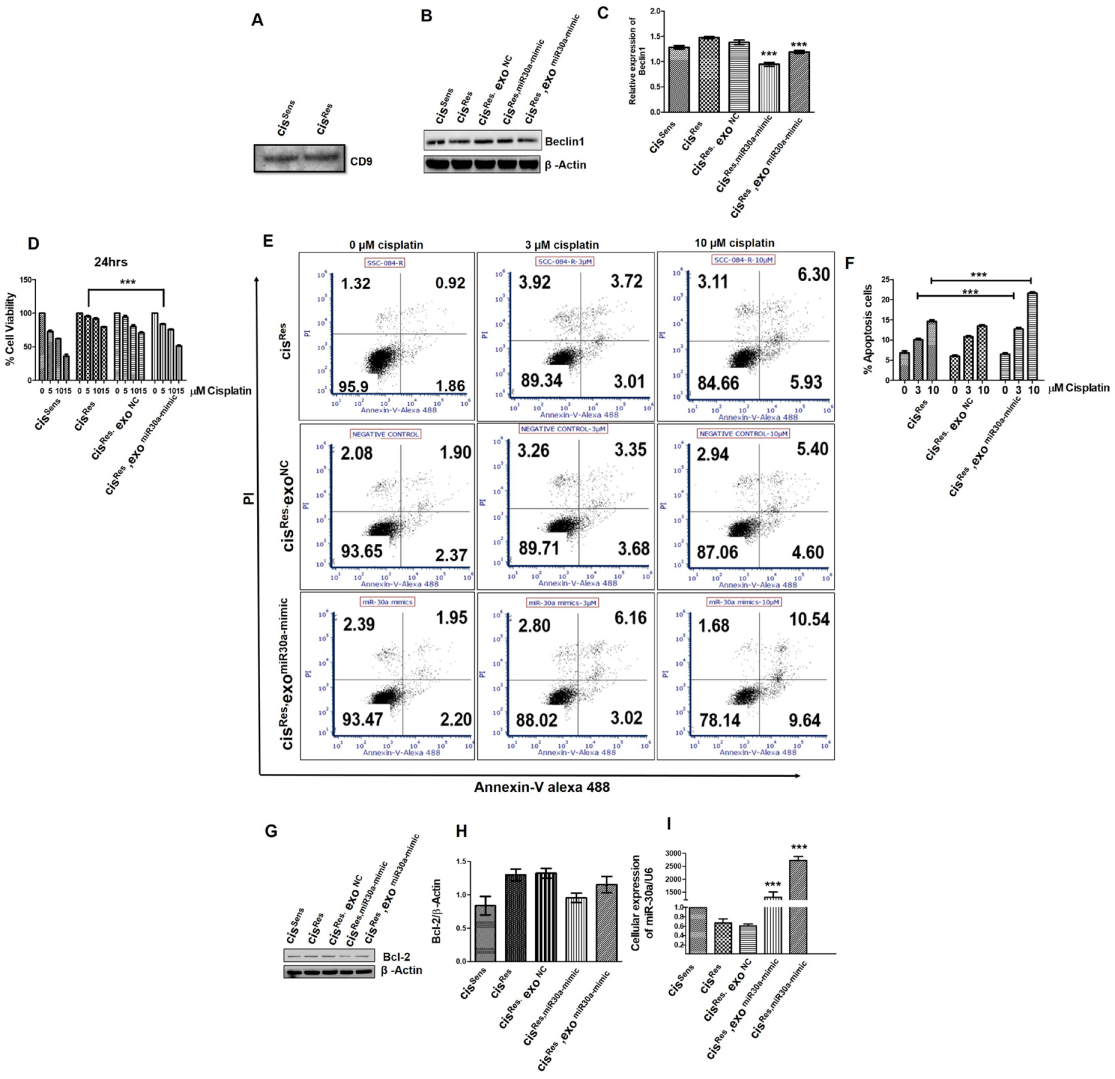
Exosomes were isolated from cis^Res^ cells transfected with either NC or miR-30a mimics and were used to treat other naïve cis^Res^ cells. cis^Sens^, cis^Res,^ and cis^Res-miR-30a mimic^ cells were employed as controls and for comparison. (**A**) The expression of CD9 in isolated exosomes from different cells was determined by Western blotting. (**B**) Beclin1 protein expression as determined by western bot. (**C**) Densitometry analysis of Beclin1 Western blot normalized to β-actin as a loading control. (**D**) Following exosomes treatment, cells were exposed to different concentrations of cisplatin (μM), as indicated) for 24 h. Cell viability was measured by the MTT assay. (**E**) For determining apoptosis, after cisplatin treatment, cells were harvested, stained with FITC-conjugated annexin-V and PI and analyzed by flow cytometry. (**F**) Graphical representation of the percentage of apoptotic cells as analyzed by FACS analysis. (**G**) BCl2 protein expression as determined by Western blot. (**H**) Densitometry analysis of BCl2 Western blot normalized to β-actin as a loading control. (**I**) miR-30a expression levels as determined by real-time PCR. Error bars represent the mean ± SD of three independent experiments. ^***^ *p* < 0.001.

Exosomes are known to play an essential role in cellular crosstalk within tumor-microenvironment [30]. In the present study, exosomes isolated from both cis^Res^ OSCC cells and serum of OSCC patients showed a marked reduction in miR-30a expression levels (Figure 2). Having observed the essential role of miR-30a restoration on the induction of apoptosis and reversal of cisplatin sensitivity in the Cis^Res^ OSCC cells (Figure 4), we next intended to determine if there is any exosomal contribution in mediating these effects. To answer this, we employed a co-conditioning approach wherein, we first restored miR-30a levels in Cis^Res^ cells using mimics (Cis^Res, miR-30a-mimic^), thereafter isolated exosomes from these cells and use the isolated exosomes to treat naïve Cis^Res^ cells (hereafter referred as cis^Res^; exo^miR-30a-mimic^) and analyzed the effects on various functional parameters. The quality of our exosomal preparation was checked and confirmed by a Western blot analysis of exosomal surface marker CD9 (Figure 6A). Notably, we found that expression of Beclin1 (a miR-30a target) is significantly decreased in the cis^Res^; exo^miR-30a-mimic^ cells compared to cis^Res^ cells. It is worth mentioning that this reduction in Beclin 1expression is comparable to the levels of Beclin1 measured in cis^Res^ following miR-30a restoration and also in the parental cis^Sens^ (Figure 6B, 6C). Furthermore, cisplatin sensitivity was significantly enhanced in the cis^Res^; exo^miR-30a-mimic^ cells when compared to the cis^Res^ OSCC cells (Figure 6D) indicating that exosomal-mediated miR-30a transfer conferred a sensitive trait. Besides, exosomal-mediated miR-30a delivery led to the induction of apoptosis in the cis^Res^; exo^miR-30a-mimic^ cells. There is an increment of Annexin V^+^PI^+^ population from 6.3 to 10.54% in cis^Res^ *vs* cis^Res^; exo^miR-30a-mimic^ cells (Figure 6E, 6F) and decreased Bcl2 expression (Figure 6G, 6H). Interestingly, miR-30a expression in the cis^Res^; exo^miR-30a-mimic^ cells were significantly enhanced when compared to cis^Res^ cells, though the extent of increase was less than that observed with the use of commercial mimics in Cis^Res, miR-30a-mimic^ (Figure 6I). These findings thus suggest that exosomes play an essential role in mediating miR-30a transfer and hence restoring cisplatin sensitivity, enhancing apoptosis and decreasing autophagic response in cis^Res^ OSCC cells.

## DISCUSSION

Exosomes, the membrane-bound extracellular vesicles, play an essential role in transferring molecular and genetic information from one cell to another [24, 31]. Some of the miRNAs they cargo serve as a critical regulator of cell-cycle, metabolism, proliferation, migration, invasion, angiogenesis and hence act as a modulator of the tumor microenvironment and chemoresistance [32, 33]. Resistance to anticancer agents is a foremost obstacle to the successful treatment of OSCC, and therefore understanding of the underlying mechanisms is essential for overcoming this therapeutic barrier. In the present study, we provide evidence that exosomal-mediated delivery of miR30a plays an essential role in reversing the sensitivity of cisplatin-resistant OSCC cells, thereby offers a potential therapeutic strategy to overcome the drug-resistance hurdle in OSCC treatment.

Aberrant expression of miRNAs has been linked with tumor progression, metastasis and chemo-resistance observed in various cancer [34]. Pioneering studies have functionally validated many of these miRNAs and demonstrated their mode of action *via* specific gene-target [35, 36]. However, there is still much remains to be done: allocate a particular exosomal-miRNA with specific cancer, identify its functional gene-target, determine its relevance in drug-resistance and finally to validate these signatures as a prognostic biomarker.

Through a combinatorial approach involving comprehensive literature search and bioinformatics web-based tools, we identified miRNAs that were either up- or down-deregulated in OSCC namely (miR-572, miR-214, miR-200a, miR-7, miR-15a, miR-155a) and (miR-30a, miR-22, miR-21, miR-19a, miR-374, miR-122a, miR-195) respectively [37, 38]. A recent *in silico* comparative profiling study documented six miRNAs that may be associated with cisplatin resistance in oral cancer (miR130-b, miR-134, miR-149, miR-491, miR-181d, miR-30a) from a total of 36 miRNAs taken into consideration [26]. Studies in renal cell carcinoma, chronic myeloid leukemia, and ovarian cancer showed a diminution in miR-30a expression facilitated apoptosis through an autophagy-reliant pathway [28]. Besides, miR-30a overexpression in lung cancer cells has been reported to enhance their paclitaxel sensitivity [27]. In contrast, miR-30a was found to be overexpressed in cisplatin-resistant melanoma cells, and therefore treatment with miR-30a inhibitor enhanced their chemo-sensitivity [39]. Thus, miR-30a may be employed to reestablish the chemo-sensitivity of certain tumor cells, however, its implication in cisplatin-induced resistance in OSCC remains unknown thus far. Not only this, but the relevance of miR-30a downregulation on various functional attributes of OSCC also remains obscure. While this manuscript was in preparation, miR-30a downregulation in OSCC was shown to suppress cell proliferation and invasion via modulating fibroblast activation protein alpha [25]. Nevertheless, its association with cisplatin-resistance and related functional effects such as autophagy, apoptosis, and migration have not yet been studied. In the present study, we conclusively present evidence demonstrating the loss of miR-30a expression in the cisplatin-resistant oral cancer cells. It is important to mention that besides observation in cisplatin resistant cells, we report here for the first time, decreased miR-30 expression in exosomes isolated from the serum of oral cancer patients vs healthy controls; the effect being more pronounced in the patients with disease recurrence post cisplatin treatment, thus substantiating the association of miR-30a with cisplatin-resistance.

miRNA functions by binding to the 3′UTR of mRNA of the target gene, thereby interfering with its translation and expression. Hence, the identification of downstream targets of miRNA is indispensable for designing efficient treatment strategies as well as overcoming drug-related resistance. For example, HOXB8 (the developmental gene) and p27 (CDKN1B, cell cycle associated gene) have been identified as the direct targets of miR-196a, and HMGA2 as a miR-204 target [40]. In line with these results, we present here original evidence that Beclin1, an autophagy-related gene, acts as the miR-30a target in OSCC and more importantly, showed Beclin1 upregulation corresponded to the development of cisplatin resistance. This is of special relevance as autophagy has been recognized as a tumor cell survival mechanism triggered following various treatment strategies, and hence contributory towards chemoresistance. Our findings thus argue for considering miR-30a target, Beclin1, as a promising therapeutic approach to circumvent cisplatin-resistance in OSCC. Remarkably, our results highlight that miR-30a overexpression in cisplatin-resistant cells led to a decrease in Beclin1 and hence autophagic response, which in turn is reflected in enhancements in apoptosis via downregulation of Bcl2 and concomitant increase in caspase3 expression. Altogether these effects resulted in regaining cisplatin sensitivity in OSCC cells. It is worth mentioning that *Beclin1* has recently been linked with OSCC progression [41, 42]. In corroboration with our findings, Zou *et al.* depicted the role of miR-30a in conferring chemosensitivity to the liver cancer cells *via* abating Beclin1-mediated autophagy [22]. The importance of our findings is strengthened by the fact that we show the major role of miR-30a in regulating two essential pathways: autophagy and apoptosis, ultimately resulting in sensitizing resistant oral cancer cells towards cisplatin, a commonly used chemotherapeutic drug.

Exosomes, the small extracellular vesicles and cargo of genetic material, proteins, and lipids, function as signaling aid between cancer and adjoining cells. Exosomes strongly influence tumor microenvironment as their uptake alter phenotypic and functional attributes of both the recipient and other cells in the neighborhood [43, 44]. Therefore, an in-depth understanding of exosomal contributions in moderating signaling networks associated with cancer progression, invasion, development of pre-metastatic niche, may identify potential targets to combat therapy resistance. A recent report revealed that exosomal-mediated shuttling of miR-155 from cancer-associated-tumor cells to neighboring cells in tumor microenvironment mediated chemoresistance in breast cancer cells [14]. Analogous to this, here we convincingly demonstrated that exosomal-delivered miR-30a is capable of transferring sensitive phenotype to the cis^Res^ OSCC cells in unification with turning on the apoptotic machinery while disabling the autophagic response.

**Figure 7:**
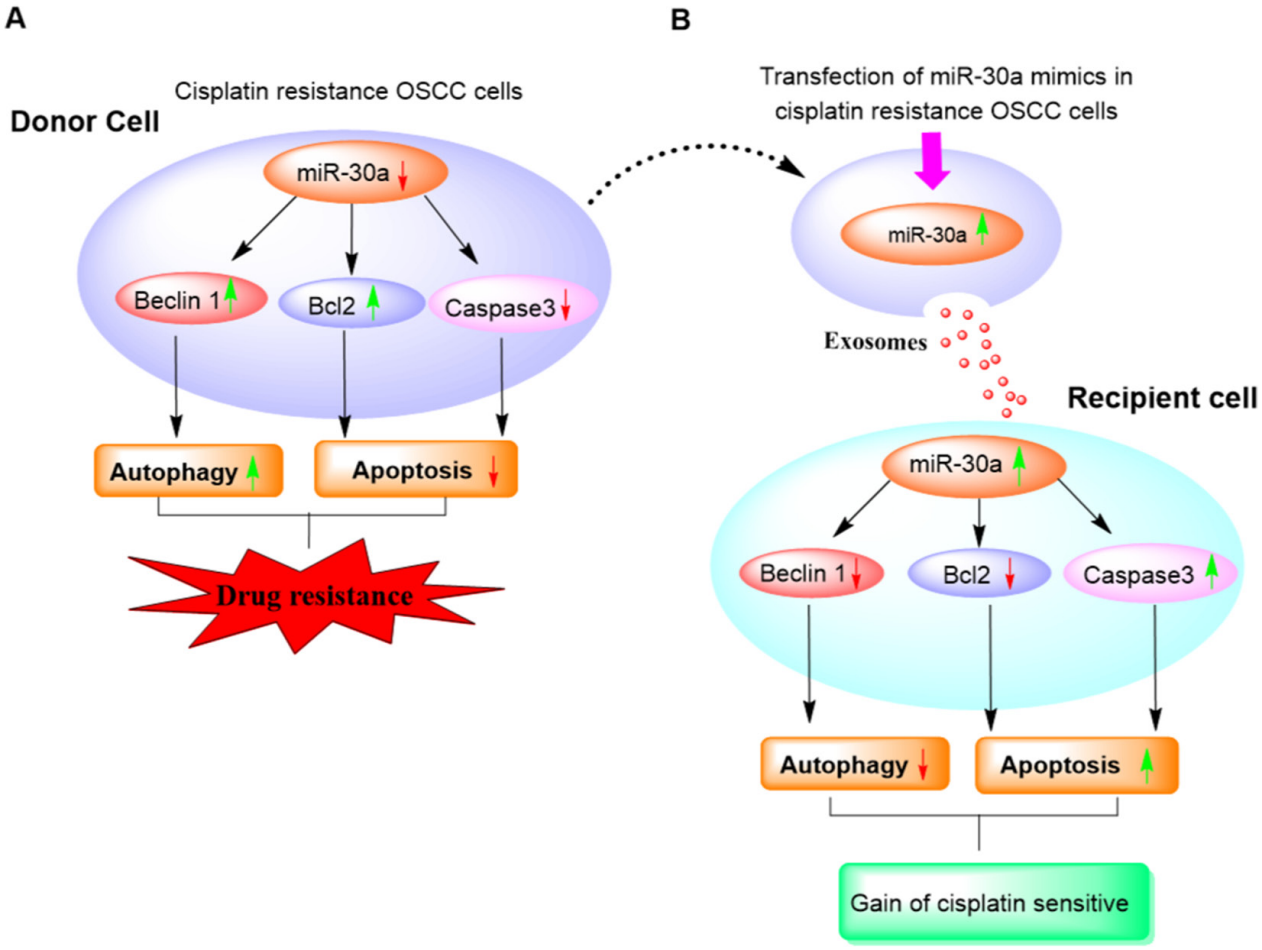
Diagram illustrating the role of exosome-mediated miR30a delivery in a reversal of cisplatin resistance in OSCC cells.

Taken together, we identified Beclin1 as the miR-30a target in oral cancer, and present here first evidence of the essential role of exosomal-miR-30a in reducing acquired chemo-resistance in OSCC *via* regulation of autophagic and apoptotic markers (Figure 7). Our study thus provides a strong rationale for employing exosomal-mediated miR-30a delivery as an effective therapeutic approach and also highlights its importance in retrieving sensitivity against cisplatin resistance, a major hurdle in conventional current approaches.

## MATERIALS AND METHODS

### miR-30a target prediction

Prediction of miRNA targets implicated in OSCC chemoresistance was done using web-based tools: TargetScan [45], DIANA-micro-T-CDS [46] and RNA-hybrid [47]. The screening of miRNA targets using TargetScan is based on the mRNA’s 3′UTR seed-match and free-energy. RNA-hybrid utilizes seed-match, free-energy, and *p-value* for predicting potential miRNAs binding sites in the mRNA’s 3′UTRs, and determines the structure with minimum free-energy. DIANA-micro-T-CDS employs an mRNA reference sequence database to determine 3′UTR sequence; dynamic algorithm programming allows the identification of minimum free-energy. We first employed TargetScan and DIANA-micro-T-CDS to predict the most potential gene targets. From the screened potential gene targets, whose involvement in the chemoresistance have been reported were further considered. Final selection was done on the basis of scores from the two tools and also based on the evaluation of binding energy as analysed by RNAhybrid.

### OSCC patients samples and cells

OSCC serum samples were provided by Dr. Rakesh Rawal, Department of Life Science, Gujarat University. The sample collection was done following the Institutional Ethics Committee of Gujarat University (GU/IEC/10/20118). Blood from healthy volunteers with or without tobacco history was collected (Table 1) and processed to obtain serum as described, with some modifications [48]. Briefly, blood samples collected in vacutainer tubes were allowed to stand at room temperature for 30–60 min to allow the clot formation. This was followed by centrifugation at 1200 g for 20 minutes at room temperature, subsequently, clear supernatant was collected in a fresh tube and stored at –80°C, until further use.

**Table 1:** Detail of clinical samples used in the study

	Variable Considered	Healthy Volunteers	Healthy Volunteers (with tobacco history)	OSCC patients	OSCC patients with tumor recurrence (post cisplatin treatment)
Gender	Number of Male / Female (Mixed)	7	7	7	6
Tobacco history	Tobacco consumption/ chewing/smoking habit	No	Yes	Yes	Yes
Tumor site		No tumor is seen	No tumor is seen	Buccal Mucosa, Tongue, and Floor of Mouth	Buccal Mucosa, Tongue, and Floor of Mouth
Tumor size	T1 & T2	0	0	5	6
	T3 & T4	0	0	2	0
Lymph node	Positive	No	No	3	
Metastasis	Positive	No	No	0	0

Human OSCC cells UPCI: SCC084 and its cisplatin-resistant strain (referred to as cis^Sens^ and cis^Res^ respectively) were received as a gift from Dr. Ruma Dey Ghosh (Tata-TCRC, Kolkata) and were cultured as previously described [26]. Briefly, both cis^Sens^ and cis^Res^ cells were grown in DMEM medium with 10% FBS, penicillin (100 units/ml), and streptomycin (100 μg/ml) at 37°C in a humidified 5% CO2 incubator. Besides, cis^Res^ cells were maintained with 1 μg/ml cisplatin (Sigma-Aldrich) for 48 h, every alternate passage. However, for experimental purposes, cis^Res^ cells were grown in the cisplatin-free culture medium.

### Western blot

The total cell lysate was prepared using RIPA buffer as described [49] and Western blotting was carried out as detailed [50]. The following antibodies were used: rabbit polyclonal anti-Beclin1 (1:2000, Abcam), Anti-Bcl2 (1:500, Abcam), Anti-CD9 antibody (1:250 Abcam) and anti-rabbit or anti-mouse secondary antibodies conjugated to horse-radish-peroxidase (1:20,000, Abcam) and β-Actin (1:5000, Santacruz Inc.,). Following TBST washes, protein bands were visualized using ECL and ChemiDoc Imaging System and quantified with Image-J.

### Exosomes isolation

Exosomes were isolated from both cell culture media (2 ml) and serum (200 μl) obtained from the clinical samples adopting the ultracentrifugation method as has been previously described with some modifications [51]. Briefly, an initial spin was performed at 300 g for 10 min at 4°C to remove the live cells. The supernatant was collected and subjected to a series of centrifugation: first at 2000 g for 10 min at 4°C to get rid of the dead cells and apoptotic bodies. The clear supernatant was collected and subjected to another round of centrifugation at 10,000 g for 30 min at 4°C. The supernatant was collected in a clean tube and ultracentrifuged for 1,00,000 g for 70 min at 4°C to obtain the exosomal pellet, which was then re-suspended in ice-cold PBS and subjected to another cycle of ultracentrifugation (as a washing step). The cleaned exosomal pellet was finally resuspended in 100 μl of PBS and stored at –80°C until further use [51].

### RNA and miRNA isolation and real-time PCR (qPCR)

Total RNA and exosomal miRNA were isolated using Qiazol and miRNeasy Kit (QIAGEN) respectively [52]. 1 μg RNA was reverse-transcribed to cDNA using iscript-cDNA synthesis kit (BioRad) and subsequently, qPCR was done using Beclin1 syber-green probe and iQ SYBR^®^ Green Supermix. 18S syber-green probe was employed as endogenous control (Supplementary Table 2). For miR-30a analysis, qPCR was performed using a TaqMan probe (AppliedBiosystems) with U6snRNA as an internal control. Results were evaluated by 2^–ΔΔCt^ quantification method [52].

### miRNA-mimics transfection

Cis^Res^ cells were transfected with either non-target control [*NTC* or Negative control (NC)] or miR-30a mimics using Lipofectamine RNAiMAX reagent as described [52]. 48 h later, cells were used in the respective experiments.

### Cell viability

Cell viability was assessed by MTT assay as described [53]. Briefly, cells in 96-well plates (1 × 10^3^ cells/well) were treated with different cisplatin concentrations. 48 h later MTT reagent was added and absorbance was read at 570 nm. The absorbance of untreated cells served as blank.

### Annexin V-PI binding

For assessing apoptosis, 0.5 × 10^6^ cells in 6-MW plates were treated with cisplatin (3 and 10 μM) for 72 h. Cells were then harvested with trypsin, washed with PBS and stained with Annexin V-Alexa 488 (Invitrogen) and PI (2 μg/ml, Sigma-Aldrich) for 15 min. Cells were analyzed using FACS S3e Cell Sorter.

### Cell migration

Cell migration was evaluated by wound healing assay as described [53]. Briefly, 0.5 × 10^6^ cells in 6-MW plates were transfected with *NC* or miR-30a mimics. 48 h later, a scratch was created using a sterile microtip. The wound area was washed gently with PBS and fresh media having different concentrations of cisplatin (0, 3 and 10 μM) was added. Photographs were taken at different times using a Zeiss inverted fluorescence microscope. The percentage of wound closure was calculated using Image J.

### Exosomes co-conditioning

To study the possible role of exosomes in the transmission of chemoresistance, 0.5 × 10^6^ cis^Res^ cells in 6-MW plates were transfected with either *NC* or miR-30a mimics. After 48 h, cells were serum-starved for 24 h and subsequently, exosomes were isolated. Exosomal preparation was used to treat naïve cis^Res^ cells for 48 h and then cellular miR-30a levels were analyzed.

### Beclin1 cloning and luciferase reporter assay

*Beclin1* 3′UTR target sequence in miR-30a was synthesized from IDT (Supplementary Table 3). The reconstituted oligonucleotides were annealed and cloned in the pmirGLO dual-luciferase vector (Promega) at the restriction-endonuclease (RE) sites (XbaI, PmeI). Following ligation, clones were transformed into E. coli DH5α cells. The plasmid was isolated, purified and quantified using nanodrop. The cloned vector was verified both by RE digestion (Supplementary Figure 1) and sequencing (Supplementary Figure 2). 3′UTRs of *Beclin1* were thus cloned downstream of the luciferase gene in the pmirGLO vector (pmirGLO-BECN1). 1 × 10^5^ cis^Res^ cells/well in the 24-MW plate were co-transfected with the pmirGLO-BECN1-vector and miR-30a or *NC* mimics using Lipofectamine-2000. 48 h later, luciferase reporter activity was assayed using a Dual-Luciferase Assay System (Promega, USA). The analysis was done following normalization with Renilla luciferase activity.

### Statistical analysis

Statistical analysis was done using GraphPad Prism 5.01. Means of all data were compared by student *t-test* and ANOVA, followed by Bonferroni’s *post-ho*c test. Data are expressed as mean ± standard-deviation. *p value* < 0.05 was considered significant. In all cases, experiments have been done three times. Representative graph or figure has been shown as two other identical experiments gave similar results.

## SUPPLEMENTARY MATERIALS



## Abbreviations

miRNA: microRNA
OSCC: oral squamous cell carcinoma
NC: negative control
NTC: non-target control
Bcl2: B-cell lymphoma 2
UTR: untranslated region
ECL: enhanced chemo luminescence
TBST: tris buffer saline-Tween 20
BCA: bichinconic acid
PI: propidium iodide
RE: restriction enzyme
FACS: fluorescence associated cell sorting
