# Supplementary material for: Exosome-mediated delivery of miR-30a sensitize cisplatin-resistant variant of oral squamous carcinoma cells via modulating Beclin1 and Bcl2
Source: Oncotarget. 2020 May 19;11(20):1832–45. doi: 10.18632/oncotarget.27557 (PMC7244014; doi:10.18632/oncotarget.27557)
Supplement: Supplementary file 1 [file oncotarget-11-1832-s001.pdf]

Exosome-mediated delivery of miR-30a sensitize cisplatin-resistant variant of oral squamous carcinoma cells via modulating Beclin1 and Bcl2

SUPPLEMENTARY MATERIALS

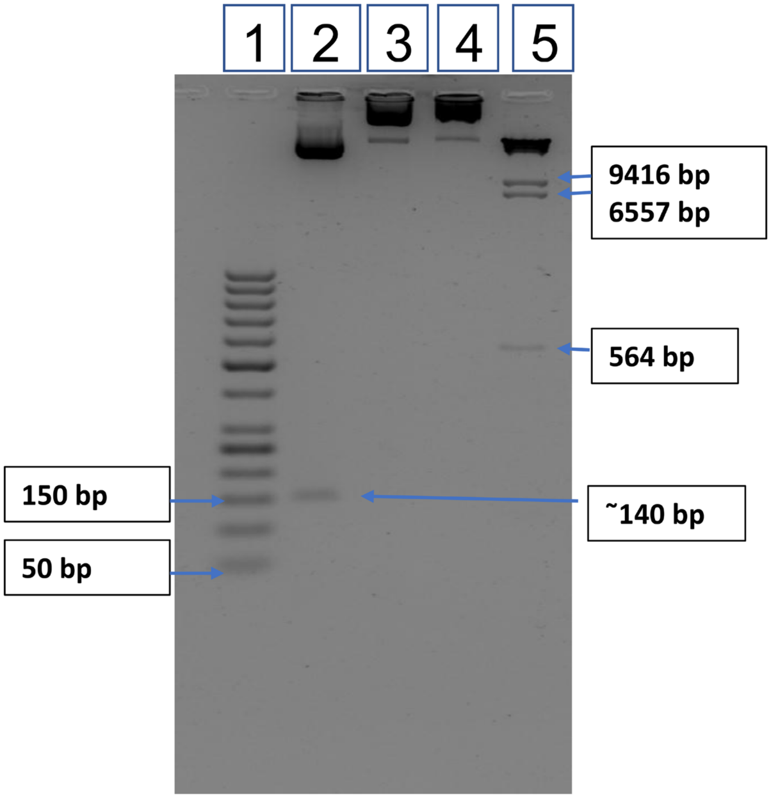

Description of lane

1 50bp ladder

2 pmirGLO vector+BECN1 digested with Not I

3 pmirGLO vector+BECN1

4 pmirGLO vector

5 *λ DNA/Hind* III ladder

Supplementary Figure 1: Confirmation of cloned vector of pmirGLO-BECN1s through restriction endonuclease digestion.

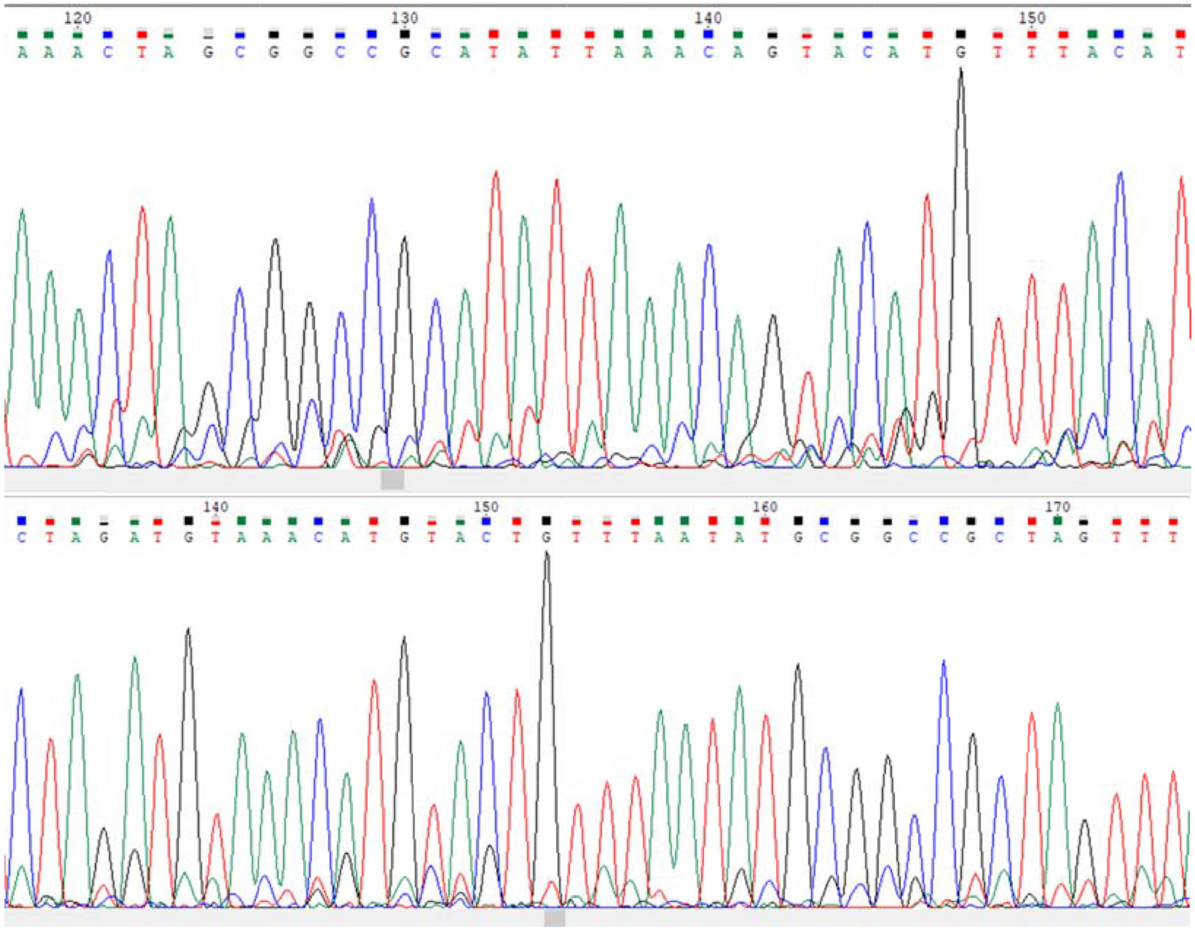

Supplementary Figure 2: Sequencing analysis of pmirGLO-BECN1 vector.

Supplementary Table 1: miR-30a target genes implicated in chemoresistance, as predicted by TargetScan and DIANA web-based tools

Gene name	Context++ score percentile	miTG SCORE
BECN1	99	0.999844
CAMK2N1	99	0.997468
ACTC1	99	0.996261
RAB32	99	0.993356
ARL4A	99	0.979199
EED	98	0.999967
SOCS1	98	0.999946
SNAIL	98	0.999918
ZNRF1	98	0.999276
PTP4A1	98	0.978827
DLGAP1	97	#
MEX3B	97	0.999923
C9orf72	97	0.999872
TWF1	97	0.997048
B3GNT5	96	0.997617
CCNE2	95	0.998636
SNX16	95	0.99759
MTDH	89	0.99991
GALNT7	87	0.999976
SCN2A	84	0.999965
TNRC6A	75	0.999923

#This gene was not predicted by DIANA web-based tools.

Supplementary Table 2: Primer sequences

Target	Primer Sequence
**18s**	**F:**5′-ATCGGGGATTGCAATTATTC-3′<br>**R:**5′-CTCACTAAACCATCCAATCG-3′
**BECN1**	**F:**5′-ACAGTGGACAGTTTGGCACA-3′<br>**R:**5′-CGGCAGCTCCTTAGATTTGT-3′

Supplementary Table 3: 3′UTR region of miR-30a targets

Targets	3′UTR Oligo
**BECN1-F**	5′-AAACTAG*CGGCCGC*ATATTAAACAGTACATGTTTACAT-3′
**BECN1-R**	3′-TTTGAT*CGCCGGCG*TATAATTTGTCATGTACAAATGTAGATC-5′
